# Interstitial Cystitis: Diagnosis and Treatment in a Pregnant Patient

**DOI:** 10.7759/cureus.14549

**Published:** 2021-04-18

**Authors:** Sofía Argüelles Rojas, José G Oviedo Ortega, Roberto Velasco Sordo

**Affiliations:** 1 Obstetrics and Gynecology, Centro Médico ABC (American British Cowdray), Mexico City, MEX

**Keywords:** interstitial cystitis, painful bladder syndrome, hyaluronic acid, intravesical treatment

## Abstract

Interstitial cystitis or painful bladder syndrome is a chronic condition characterized by severe and acyclic pelvic pain lasting for a period of at least six weeks. Although this condition is not accompanied by urinary infection, the patient’s daily activities are impeded. The most common symptoms are urinary frequency, dysuria, suprapubic pain, nycturia, and dyspareunia. The etiology of interstitial cystitis is unclear, and its diagnosis is infrequent because of the low number of cases. A definitive diagnosis is based on cystoscopic findings and typical histopathological evidence, such as Hunner’s ulcers. Herein, we describe the diagnosis and treatment of a clinical case of interstitial cystitis in a patient who started presenting symptoms during pregnancy. A 42-year-old woman at 27.2 weeks of pregnancy began showing symptoms at 10 weeks of gestation. She presented with dysuria and hypogastric pain with an intensity of 9/10, which hindered her daily activities. Physical examination revealed tenderness to deep and superficial hypogastric palpation. Routine urinalysis and urine culture test yielded negative results. She was started on symptomatic treatment from 10 weeks of gestation, but it did not result in any improvement. Therefore, intraoperative diagnostic cystoscopy was performed to obtain biopsy samples. Histopathological analysis of the samples showed evidence of interstitial cystitis. Accordingly, she was started on intravesical instillation of hyaluronic acid, which improved her condition. On the basis of the case findings, we recommend that interstitial cystitis should be considered a differential diagnosis in patients with pelvic pain and urinary symptoms unrelated to a urinary infection.

## Introduction

Interstitial cystitis or painful bladder syndrome is defined as an unpleasant sensation that can present as pain, pressure, or vesical discomfort associated with urinary symptoms that persist for more than six weeks, without any infection or other identifiable cause [1]. The pathophysiology of interstitial cystitis remains unknown because of its rarity, but its prevalence is higher in women; it can present with diverse symptoms, such as pain, pressure or discomfort in the pelvic region, and urinary frequency and urgency [2].

Moreover, this condition can present with a wide spectrum of symptoms, causing diagnostic challenges. Therefore, adequate knowledge about the clinical history, thorough physical examination, routine urinalysis, and urine culture test are very important to exclude other pathologies. Furthermore, cystoscopy with biopsy is required to confirm the diagnosis [3]. 

Many treatments are available for interstitial cystitis, and these depend on the severity of the clinical symptoms and the patients´ response. The symptoms can also be controlled via lifestyle changes, the use of analgesics and neuromodulators, or the use of intravesical medications, such as hyaluronic acid, which has recently been shown to produce good results in the management of this pathology. In particular, the use of intravesical hyaluronic acid reduces the severity of symptoms, suggesting its utility as an excellent therapeutic option [4].

We report the case of a woman who presented with clinical symptoms of interstitial cystitis, namely, severe pelvic pain, dysuria, and absence of urinary infection, which started during pregnancy. This case highlights the importance of knowledge about the pathology and symptoms, especially in patients who do not show improvements when administered symptomatic treatment.

## Case presentation

The patient described herein provided informed consent, and the report was approved by the appropriate ethics review board. The patient was a 42-year-old woman with a family history of type 2 diabetes mellitus and systemic arterial hypertension. She had no chronic degenerative illnesses but was a social smoker for 10 years, a social drinker, and allergic to metronidazole. She also had a history of pyelonephritis (first in 2017 and later in 2019 during the first trimester of her second pregnancy). Her surgical history was positive for a laparotomy appendectomy (1999) and a cesarean section (2017), both uncomplicated. 

She presented for medical consultation at 10 weeks of gestation with complaints of pelvic pain, specifically cramping, with an intensity of 7/10, as well as dysuria and intense hypogastric pain. A physical examination revealed tenderness to superficial and deep palpation in hypogastrium. Examination using a vaginal mirror revealed a closed cervix without transvaginal bleeding. Obstetric ultrasonography revealed a single, live intrauterine fetus with a fetal cardiac frequency of 154 bpm. Routine urinalysis showed cloudy urine, with a protein level of 100 mg/dL, hemoglobin level ≥ 1.0 mg/dL, leukocyte count of 9,695/µL, erythrocyte count of 1,073/µL, epithelial cell count of 13/µL, and a negative urinary culture test. These findings prompted the consideration of interstitial cystitis as a possible diagnosis and the patient was started on symptomatic treatment. 

However, at 27.2 weeks of gestation, she was admitted with hypogastric pain (intensity, 9/10). The single, live intrauterine fetus had a cardiac frequency of 143 bpm, and no changes were observed in the placenta or amniotic fluid. At this time, it was agreed to perform a diagnostic cystoscopy for further evaluation, given the patient's increasing pelvic pain. Diagnostic cystoscopy was performed and it revealed that the vesical floor, walls, and ceiling were edematous with white patches and trabeculae. Biopsy samples of the vesical floor, ceiling, and left and right walls were collected and sent for histopathological analysis to determine the definitive diagnosis. 

Subsequently, a Foley catheter was inserted, and 40 mg of intravesical hyaluronic acid was instilled. Thereafter, the catheter was withdrawn. The histopathological analysis revealed acute and chronic cystitis that presented with ulcerated, granulated tissue, and reactive atypia.

Under a diagnosis of interstitial cystitis during pregnancy, intravesical treatment with hyaluronic acid was continued with one weekly dose (instillation of 40 mg) for four weeks, followed by one monthly dose for two months. This treatment resulted in a partial improvement in the patient’s condition. The pregnancy ended at 35.2 weeks of gestation, due to the exacerbation of pelvic pain (intensity 10/10), which was disabling, and uterine activity that started. A male newborn was obtained by c-section, weight 2,045 grams, height 46 centimeters, Apgar 9/9, Silverman 1. At this time, the patient had received six intravesical doses of hyaluronic acid. 

**Figure 1:**
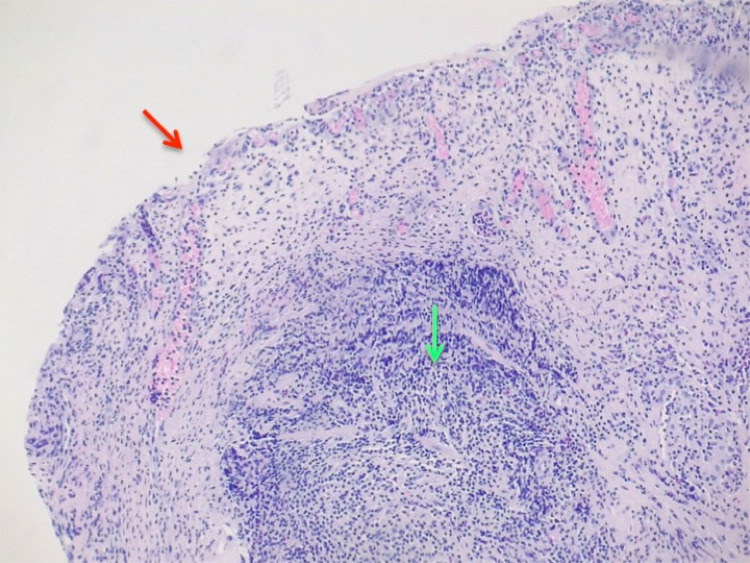
The urothelium is absent (red arrow), and a dense infiltrate containing lymphocytes and plasma cells is observed in the lamina propria (green arrow).

**Figure 2:**
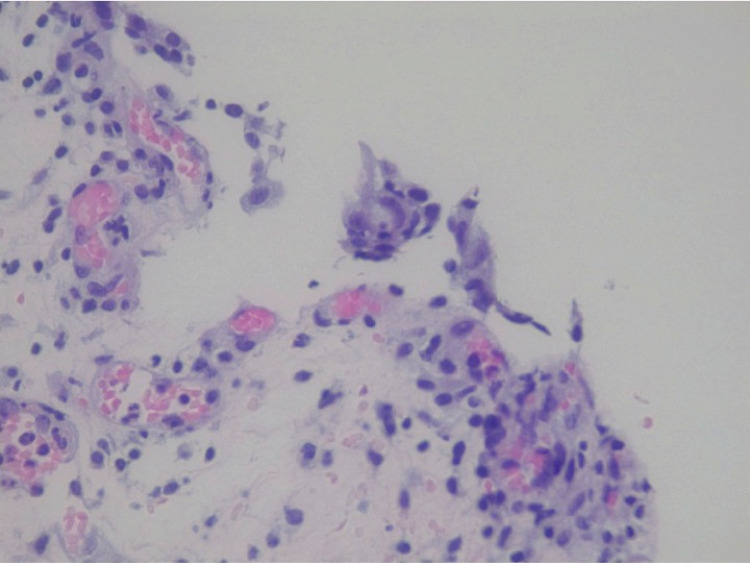
A rupture in the lamina propria.

**Figure 3:**
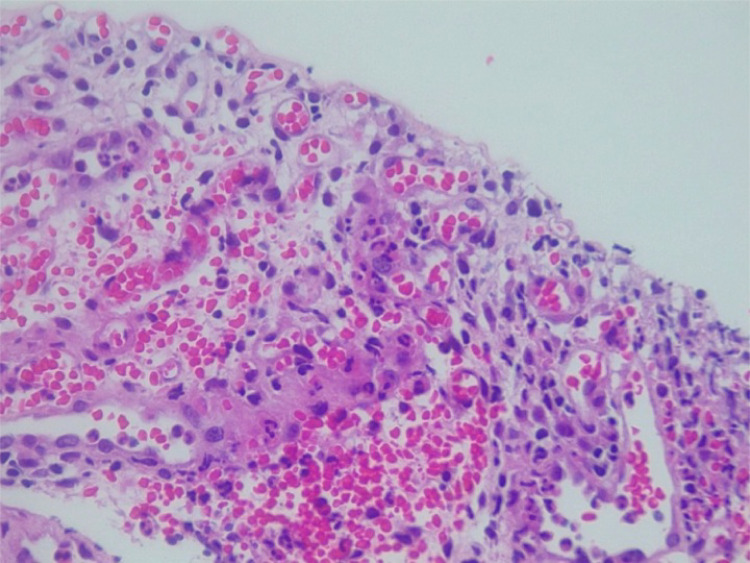
The lamina propria shows prominent vascularity.

The patient continued to experience symptoms after puerperium. Therefore, another cystoscopy was performed to obtain additional biopsy samples. Histopathological analysis of the new samples revealed acute and intense ulcerated cystitis with stromal regenerative changes and edema. The urothelium was absent, and a dense infiltrate containing lymphocytes and plasma cells was observed in the lamina propria (Figure 1). Moreover, the lamina propria appeared ruptured (Figure 2) and showed prominent vascularity (Figure 3). Accordingly, we decided to continue the treatment using intravesical hyaluronic acid and added four more monthly doses. The patient initially received a weekly dose for four weeks and one monthly dose for two months, and after pregnancy, she received an additional monthly dose for four months. This treatment improved her main symptom of vesical pain, and she could resume her daily activities. At present, the patient is asymptomatic.

## Discussion

Interstitial cystitis or painful bladder syndrome is a rare condition, difficult to diagnose and treat. It is a chronic disease characterized by severe and chronic pelvic pain of an acyclic nature lasting for a minimum of six weeks, without any urinary infection. The pain generally intensifies during menstruation and vesical fullness and diminishes with bladder emptying [5]. The most common symptoms are urinary frequency (70%), dysuria (52%), urgency (50%), suprapubic pain (50%), nycturia (35%), and dyspareunia (13%) [6]. The incidence is 15 per 100,000 women per year [6]. The RICE study reported a prevalence of 3.3 to 7.9 million (2.7-6.5%) among women aged 18 years and older in the USA [2]. The female:male ratio of prevalence is 5:1, and the average age of incidence is 51 years (range, 31-81 years) [6].

Interstitial cystitis characterized by symptoms appearing during pregnancy only occurs in 7% of pregnant women. The remaining cases of interstitial cystitis occur before pregnancy, after the first pregnancy, or during puerperium (first six months) [7]. The etiology of the disease remains unknown, but various theories have been proposed regarding the pathophysiology of the condition. It is considered a disorder of the urothelium due to defects in the vesical mucous membrane, which secretes glycosaminoglycans, and the urinary tissue barrier. Patients with interstitial cystitis show a decrease in the concentration of hyaluronic acid and increasing potassium permeability in the urothelium, thereby aggravating bladder pain. This is demonstrated principally by a positive potassium sensitivity test and a good response to the glycosaminoglycan restoration agent, that is, intravesical hyaluronic acid.

In pregnant women, as in our present case, the most accepted theory is an alteration in the permeability of the urothelium, which affects its function as a protective barrier. Fluctuations in estrogen and progesterone levels during pregnancy stimulate the growth of urothelial cells. Similarly, estrogen and progesterone influence growth factors, such as the nerve growth factor, epidermal growth factor, and heparin-binding growth factor. All of these factors are present in the urine or bladder of patients with interstitial cystitis. Another theory proposes the activation of the mast cells in the bladder by estrogen, resulting in the release of histamines and other mediators that cause pain and inflammation [8].

The diagnosis and treatment of interstitial cystitis remain a challenge because of the lack of research, low number of cases, and wide range of symptoms varying from patient to patient. Therefore, diagnosis is generally time-consuming. Because of these difficulties, patients are often referred to a diverse range of specialists, including family physicians, gynecologists, urologists, and pain specialists, which adds to the patient’s confusion and frustration [5].

Hence, the role of gynecologists in diagnosing this condition is fundamental, as they are often the primary physician for many young women. Moreover, gynecologists should consider interstitial cystitis as a differential diagnosis in patients who present with pelvic pain, especially if it is accompanied by other urinary symptoms but without any evidence of an infection [6].

In most cases, interstitial cystitis is a diagnosis of exclusion, therefore, adequate evaluation is necessary to exclude other conditions that may be causing the vesical irritation symptoms. A pelvic examination must be performed, together with routine urinalysis and cystoscopy. Cystoscopy is essential because the definitive diagnosis is based on the findings and typical histopathological features of the condition, such as Hunner’s ulcers [5].

The treatment of interstitial cystitis or painful bladder syndrome also presents significant difficulties because of the lack of knowledge about the etiology of the condition. The symptoms vary considerably among patients, the definitions of the illness are different, and the therapeutic results are not always the same. In addition, only a few good-quality studies (randomized trials) have investigated the effectiveness and safety of various treatments for this condition [9].

Glycosaminoglycans are commonly used for treating this condition. They are classified into four families: heparin and heparan sulfate, chondroitin and dermatan sulfate, hyaluronan, and keratan sulfate [4]. At present, hyaluronic acid and chondroitin sulfate are the glycosaminoglycans most used in intravesical treatment. A study that compared different intravesical treatments with different agents, instillation protocols, and pain scales showed that symptoms improved in all cases. However, the studies that showed the greatest reduction in symptoms (Cohen’s d > 2) were those that used high molecular weight hyaluronic acid, which is also more cost-effective than other intravesical agents [4]. 

In this case, the diagnostic protocol involved a pelvic examination, routine urinalysis, and cystoscopy. A definitive diagnosis was obtained on the basis of the results of vesical biopsies, which are not contraindicated during pregnancy. The treatment was performed using hyaluronic acid, which is also not contraindicated in pregnant women and poses a risk of fetal deformity in only 2-4% of cases [7]. The patient received six doses of hyaluronic acid during pregnancy and four doses during puerperium. Although the symptoms improved significantly, the improvement was not immediate because glycosaminoglycan regeneration in the urothelium is not immediate, but gradual instead. 

The recommended treatment plan with intravesical hyaluronic acid is 40 mg/week for four weeks. Thereafter, monthly doses should be administered for 12 months. The duration of the instillations can be varied from six weeks to 12 months, with a final evaluation period of symptoms between three months and five years [4].

In the present case, the patient showed a partial improvement of symptoms with six doses (i.e., three months of treatment), and subsequent re-intervention was required only for exacerbated symptoms. The treatment was continued with four monthly doses until 10 doses were completed (i.e., seven months of treatment). The improvement in symptoms, mainly the reduction in vesical pain after seven months of treatment, is described as the range for the final evaluation. 

## Conclusions

Interstitial cystitis is a chronic condition characterized by severe, acyclic pelvic pain and urinary symptoms without any evidence of a urinary infection. Accurate diagnosis and treatment of interstitial cystitis require a thorough assessment of the symptoms, as well as their duration. In this case, the characteristics of the symptoms, without any evidence of a urinary infection, led us to the suspicion of interstitial cystitis. Study protocol includes cystoscopy and histopathological analysis for confirmation of definitive diagnosis. Based on our case, we believe treatment options should include intravesical glycosaminoglycans administration, specifically high molecular weight hyaluronic acid should be considered. More studies are required to further understand the pathophysiology of this rare condition and its treatment.
